# Supplementary material for: The Arabidopsis J-Protein AtDjC5 Facilitates Thermotolerance Likely by Aiding in the ER Stress Response
Source: Int J Mol Sci. 2022 Oct 28;23(21):13134. doi: 10.3390/ijms232113134 (PMC9654137; doi:10.3390/ijms232113134)
Supplement: Supplementary file 1 [file ijms-23-13134-s001.zip › ijms-1888802-supplementary.pdf]

Supporting information

Table S1. Primers for real-time PCR amplification

Target genes		Primer sequences (from 5′ to 3′)
AtJ5	FP	GACGCTGGAGGCAGTCACTAT
	RP	CCGCTGTTGTTGGATTTTCC
BIP1	FP	GGGAGAGATTAAGTAACGTC
	RP	ACTTACTCTTTAAAAACTTTATCG
BIP2	FP	GAGAGATTAAGTAGCGTCCCT
	RP	AAAGGTATTTGTTATGAAGAAGT
BIP3	FP	ACAGCGAAGATGACGAGGA
	RP	AACTTGACATCAAAGCTGCTC
BZIP17	FP	CAAGCTTGTGAAGATAGATGGGA
	RP	TAGAGGCAGTGCAGGGGTAT
BZIP28	FP	ATCCTAAGCCTGTCTCGAGTTGTA
	RP	CGCCGACCATTAAAACCCTC
BZIP60	FP	CGATGATGCTGTGGCTAAAA
	RP	TCTCAAGCATTCTCTTTCGAGAT

Table S2. Primers for cloning of *AtJ5*-specific amiRNAs

amiRNA mutant	Primer sequences (from 5´ to 3´)
atj5-2	FP: GGATCCCTGCAGCCCCAAACACACG
	RP: GAATTCCCCCATGGCGATGCCTTAAA
	I: gaTAAGCAAGATAGTTACTGCCTtctctcttttgtattcc
	II: gaAGGCAGTAACTATCTTGCTTAtcaaagagaatcaatga
	III: gaAGACAGTAACTATGTTGCTTTtcacaggtcgtgatatg
	IV: gaAAAGCAACATAGTTACTGTCTtctacatatatattcct
atj5-3	FP: GGATCCCTGCAGCCCCAAACACACG
	RP: GAATTCCCCCATGGCGATGCCTTAAA
	I: gaTTCTGGGTGATTTACAACCATtctctcttttgtattcc
	II: gaATGGTTGTAAATCACCCAGAAtcaaagagaatcaatga
	III: gaATAGTTGTAAATCTCCCAGATtcacaggtcgtgatatg
	IV: gaATCTGGGAGATTTACAACTATtctacatatatattcct
